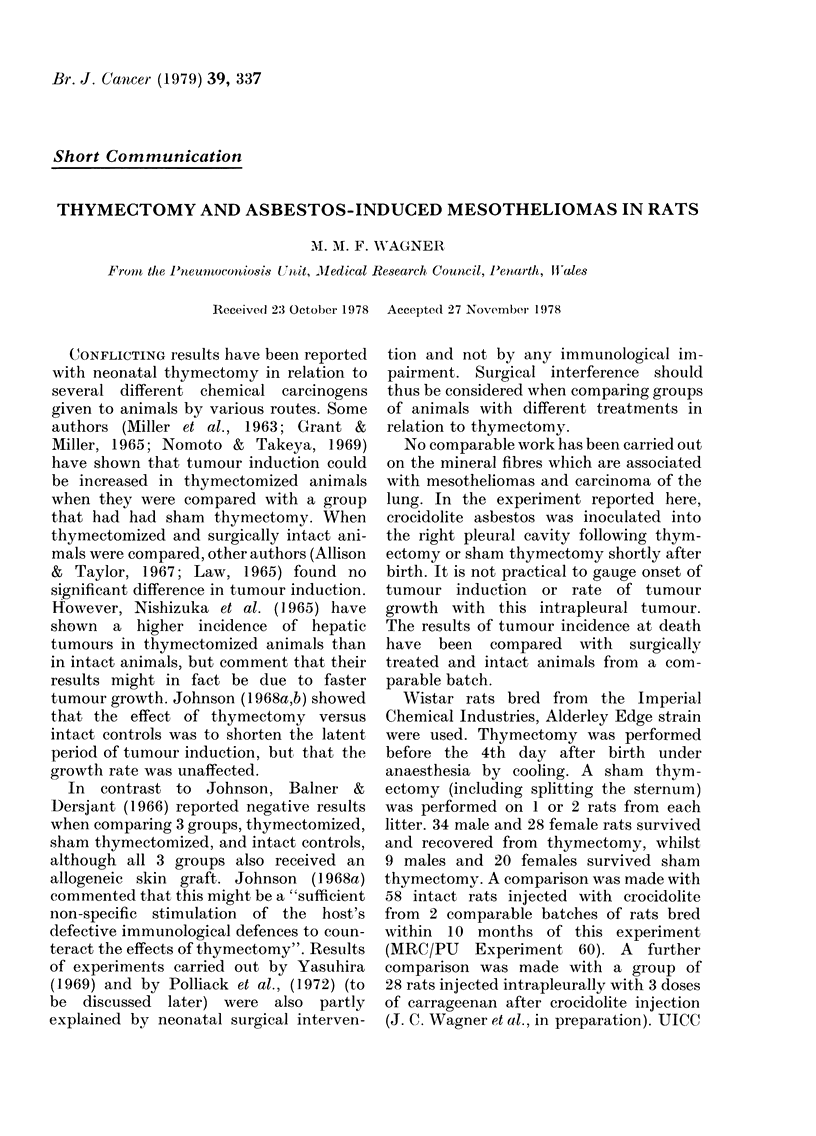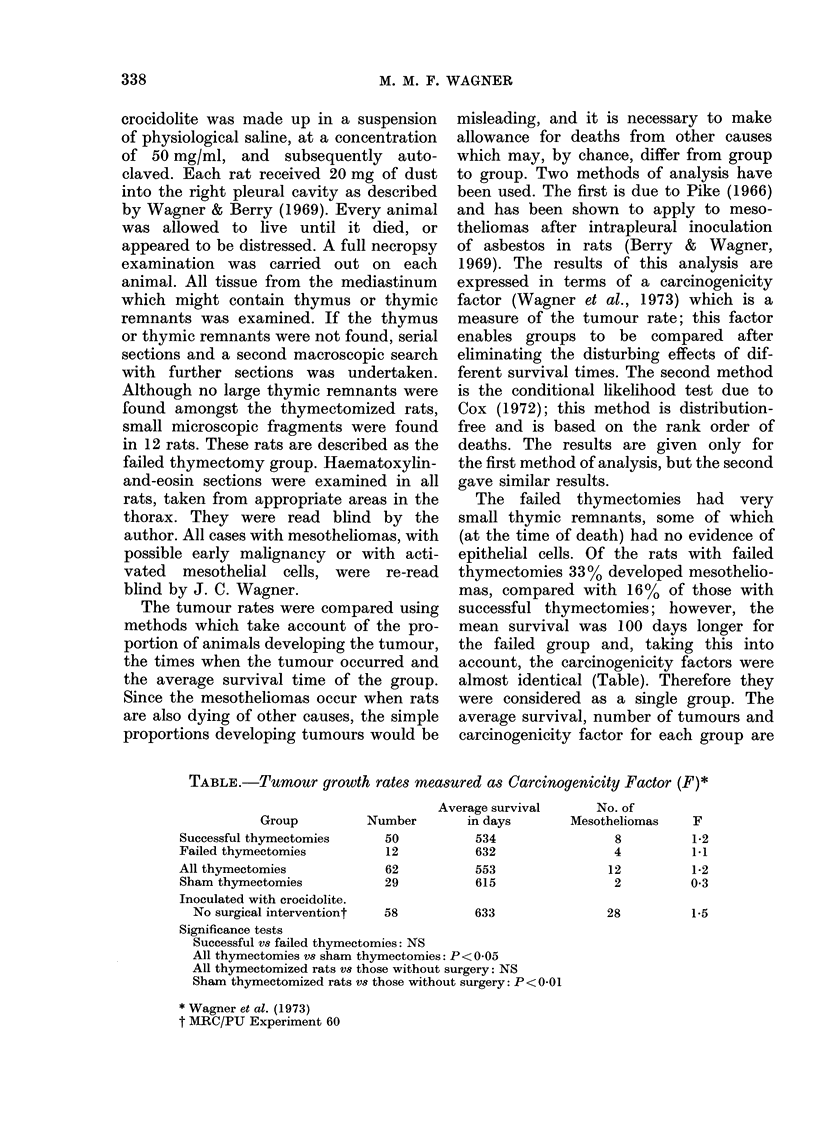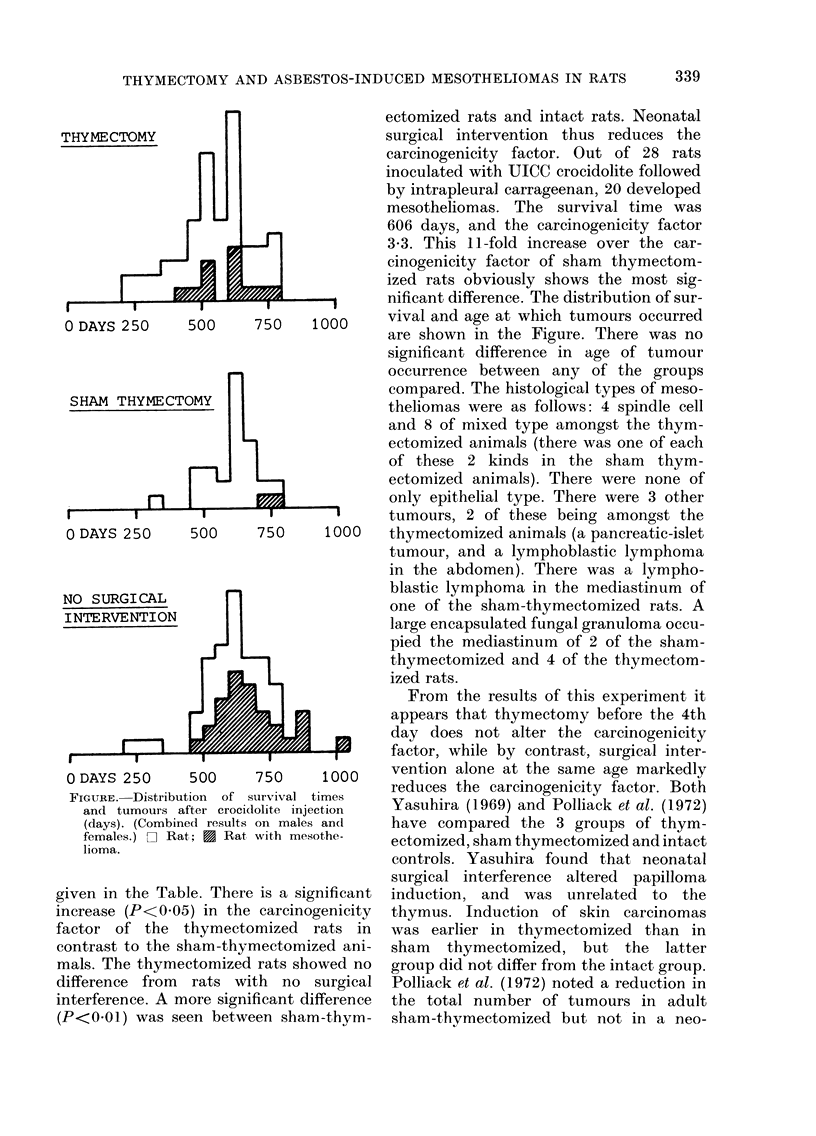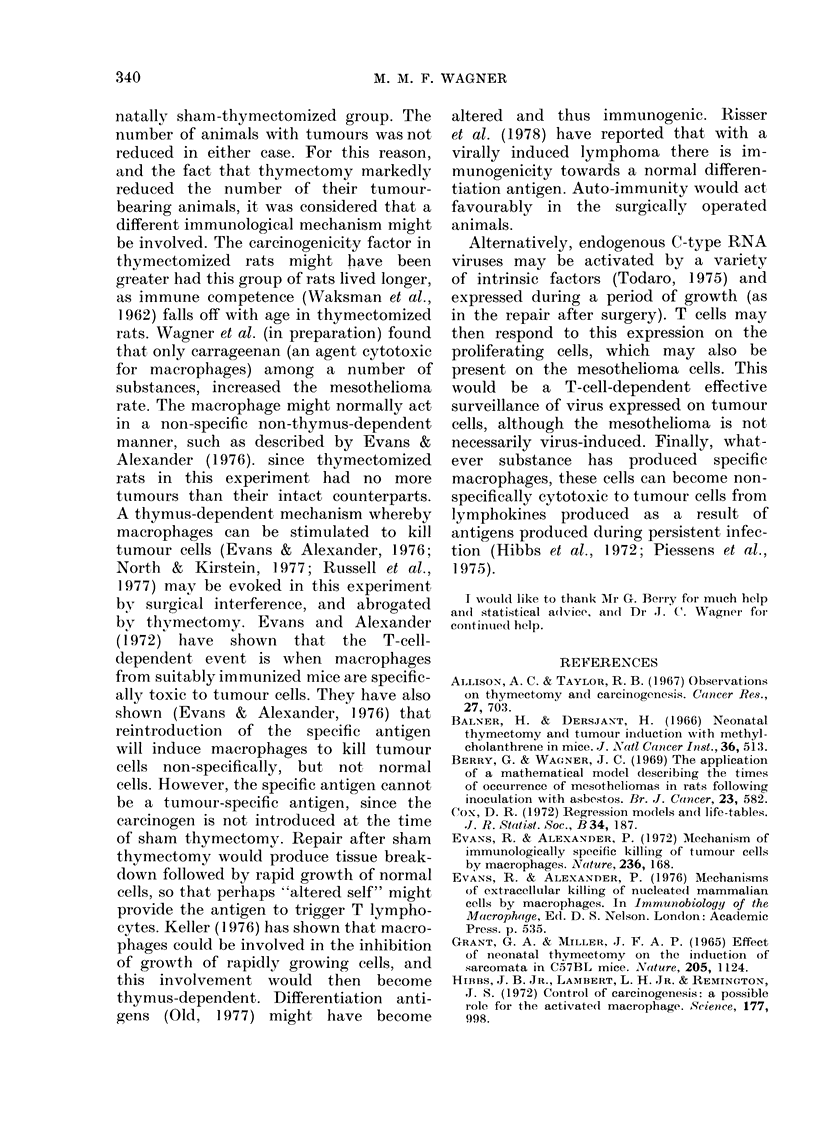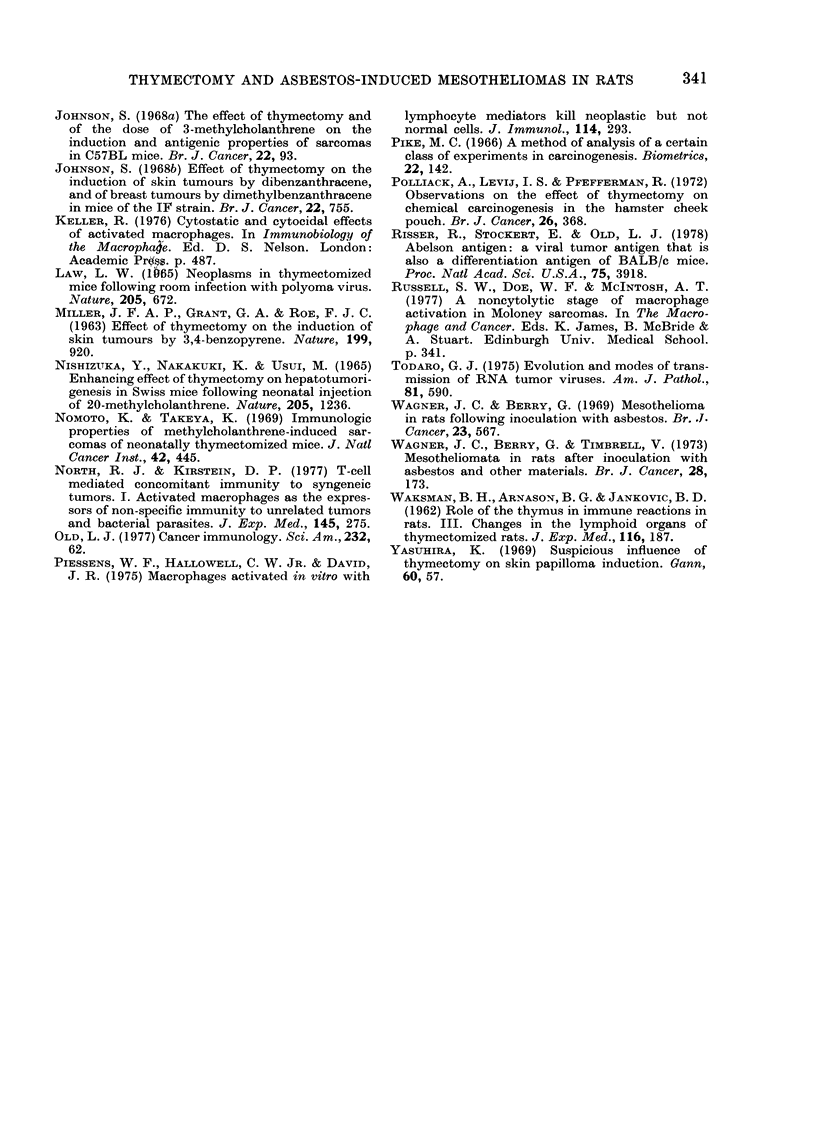# Thymectomy and asbestos-induced mesotheliomas in rats.

**DOI:** 10.1038/bjc.1979.58

**Published:** 1979-03

**Authors:** M. M. Wagner


*Br. J. Cancer* (1979) **39**, 337

***Short Communication***

### THYMECTOMY AND ASBESTOS-INDUCED MESOTHELIOMAS IN RATS

M. M. F. WAGNER

*From the Pneumoconiosis Unit, Medical Research Council, Penarth, Wales*

Received 23 October 1978 Accepted 27 November 1978

CONFLICTING results have been reported with neonatal thymectomy in relation to several different chemical carcinogens given to animals by various routes. Some authors (Miller *et al.*, 1963; Grant & Miller, 1965; Nomoto & Takeya, 1969) have shown that tumour induction could be increased in thymectomized animals when they were compared with a group that had had sham thymectomy. When thymectomized and surgically intact animals were compared, other authors (Allison & Taylor, 1967; Law, 1965) found no significant difference in tumour induction. However, Nishizuka *et al.* (1965) have shown a higher incidence of hepatic tumours in thymectomized animals than in intact animals, but comment that their results might in fact be due to faster tumour growth. Johnson (1968*a*,*b*) showed that the effect of thymectomy versus intact controls was to shorten the latent period of tumour induction, but that the growth rate was unaffected.

In contrast to Johnson, Balner & Dersjant (1966) reported negative results when comparing 3 groups, thymectomized, sham thymectomized, and intact controls, although all 3 groups also received an allogeneic skin graft. Johnson (1968*a*) commented that this might be a "sufficient non-specific stimulation of the host's defective immunological defences to counteract the effects of thymectomy". Results of experiments carried out by Yasuhira (1969) and by Polliack *et al.*, (1972) (to be discussed later) were also partly explained by neonatal surgical intervention and not by any immunological impairment. Surgical interference should thus be considered when comparing groups of animals with different treatments in relation to thymectomy.

No comparable work has been carried out on the mineral fibres which are associated with mesotheliomas and carcinoma of the lung. In the experiment reported here, crocidolite asbestos was inoculated into the right pleural cavity following thymectomy or sham thymectomy shortly after birth. It is not practical to gauge onset of tumour induction or rate of tumour growth with this intrapleural tumour. The results of tumour incidence at death have been compared with surgically treated and intact animals from a comparable batch.

Wistar rats bred from the Imperial Chemical Industries, Alderley Edge strain were used. Thymectomy was performed before the 4th day after birth under anaesthesia by cooling. A sham thymectomy (including splitting the sternum) was performed on 1 or 2 rats from each litter. 34 male and 28 female rats survived and recovered from thymectomy, whilst 9 males and 20 females survived sham thymectomy. A comparison was made with 58 intact rats injected with crocidolite from 2 comparable batches of rats bred within 10 months of this experiment (MRC/PU Experiment 60). A further comparison was made with a group of 28 rats injected intrapleurally with 3 doses of carrageenan after crocidolite injection (J. C. Wagner *et al.*, in preparation). UICC

crocidolite was made up in a suspension of physiological saline, at a concentration of 50 mg/ml, and subsequently autoclaved. Each rat received 20 mg of dust into the right pleural cavity as described by Wagner & Berry (1969). Every animal was allowed to live until it died, or appeared to be distressed. A full necropsy examination was carried out on each animal. All tissue from the mediastinum which might contain thymus or thymic remnants was examined. If the thymus or thymic remnants were not found, serial sections and a second macroscopic search with further sections was undertaken. Although no large thymic remnants were found amongst the thymectomized rats, small microscopic fragments were found in 12 rats. These rats are described as the failed thymectomy group. Haematoxylin-and-eosin sections were examined in all rats, taken from appropriate areas in the thorax. They were read blind by the author. All cases with mesotheliomas, with possible early malignancy or with activated mesothelial cells, were re-read blind by J. C. Wagner.

The tumour rates were compared using methods which take account of the proportion of animals developing the tumour, the times when the tumour occurred and the average survival time of the group. Since the mesotheliomas occur when rats are also dying of other causes, the simple proportions developing tumours would be misleading, and it is necessary to make allowance for deaths from other causes which may, by chance, differ from group to group. Two methods of analysis have been used. The first is due to Pike (1966) and has been shown to apply to mesotheliomas after intrapleural inoculation of asbestos in rats (Berry & Wagner, 1969). The results of this analysis are expressed in terms of a carcinogenicity factor (Wagner *et al.*, 1973) which is a measure of the tumour rate; this factor enables groups to be compared after eliminating the disturbing effects of different survival times. The second method is the conditional likelihood test due to Cox (1972); this method is distribution-free and is based on the rank order of deaths. The results are given only for the first method of analysis, but the second gave similar results.

The failed thymectomies had very small thymic remnants, some of which (at the time of death) had no evidence of epithelial cells. Of the rats with failed thymectomies 33% developed mesotheliomas, compared with 16% of those with successful thymectomies; however, the mean survival was 100 days longer for the failed group and, taking this into account, the carcinogenicity factors were almost identical (Table). Therefore they were considered as a single group. The average survival, number of tumours and carcinogenicity factor for each group are

TABLE.—*Tumour growth rates measured as Carcinogenicity Factor (F)**

| Group | Number | Average survival in days | No. of Mesotheliomas | F |
|---|---|---|---|---|
| Successful thymectomies | 50 | 534 | 8 | 1·2 |
| Failed thymectomies | 12 | 632 | 4 | 1·1 |
| All thymectomies | 62 | 553 | 12 | 1·2 |
| Sham thymectomies | 29 | 615 | 2 | 0·3 |
| Inoculated with crocidolite. No surgical intervention† | 58 | 633 | 28 | 1·5 |

Significance tests
Successful *vs* failed thymectomies: NS
All thymectomies *vs* sham thymectomies: $P < 0{\cdot}05$
All thymectomized rats *vs* those without surgery: NS
Sham thymectomized rats *vs* those without surgery: $P < 0{\cdot}01$

* Wagner *et al.* (1973)

† MRC/PU Experiment 60

FIGURE.—Distribution of survival times and tumours after crocidolite injection (days). (Combined results on males and females.) □ Rat; ▨ Rat with mesothelioma.

given in the Table. There is a significant increase ($P<0{\cdot}05$) in the carcinogenicity factor of the thymectomized rats in contrast to the sham-thymectomized animals. The thymectomized rats showed no difference from rats with no surgical interference. A more significant difference ($P<0{\cdot}01$) was seen between sham-thymectomized rats and intact rats. Neonatal surgical intervention thus reduces the carcinogenicity factor. Out of 28 rats inoculated with UICC crocidolite followed by intrapleural carrageenan, 20 developed mesotheliomas. The survival time was 606 days, and the carcinogenicity factor 3·3. This 11-fold increase over the carcinogenicity factor of sham thymectomized rats obviously shows the most significant difference. The distribution of survival and age at which tumours occurred are shown in the Figure. There was no significant difference in age of tumour occurrence between any of the groups compared. The histological types of mesotheliomas were as follows: 4 spindle cell and 8 of mixed type amongst the thymectomized animals (there was one of each of these 2 kinds in the sham thymectomized animals). There were none of only epithelial type. There were 3 other tumours, 2 of these being amongst the thymectomized animals (a pancreatic-islet tumour, and a lymphoblastic lymphoma in the abdomen). There was a lymphoblastic lymphoma in the mediastinum of one of the sham-thymectomized rats. A large encapsulated fungal granuloma occupied the mediastinum of 2 of the sham-thymectomized and 4 of the thymectomized rats.

From the results of this experiment it appears that thymectomy before the 4th day does not alter the carcinogenicity factor, while by contrast, surgical intervention alone at the same age markedly reduces the carcinogenicity factor. Both Yasuhira (1969) and Polliack *et al.* (1972) have compared the 3 groups of thymectomized, sham thymectomized and intact controls. Yasuhira found that neonatal surgical interference altered papilloma induction, and was unrelated to the thymus. Induction of skin carcinomas was earlier in thymectomized than in sham thymectomized, but the latter group did not differ from the intact group. Polliack *et al.* (1972) noted a reduction in the total number of tumours in adult sham-thymectomized but not in a neonatally sham-thymectomized group. The number of animals with tumours was not reduced in either case. For this reason, and the fact that thymectomy markedly reduced the number of their tumour-bearing animals, it was considered that a different immunological mechanism might be involved. The carcinogenicity factor in thymectomized rats might have been greater had this group of rats lived longer, as immune competence (Waksman *et al.*, 1962) falls off with age in thymectomized rats. Wagner *et al.* (in preparation) found that only carrageenan (an agent cytotoxic for macrophages) among a number of substances, increased the mesothelioma rate. The macrophage might normally act in a non-specific non-thymus-dependent manner, such as described by Evans & Alexander (1976). since thymectomized rats in this experiment had no more tumours than their intact counterparts. A thymus-dependent mechanism whereby macrophages can be stimulated to kill tumour cells (Evans & Alexander, 1976; North & Kirstein, 1977; Russell *et al.*, 1977) may be evoked in this experiment by surgical interference, and abrogated by thymectomy. Evans and Alexander (1972) have shown that the T-cell-dependent event is when macrophages from suitably immunized mice are specifically toxic to tumour cells. They have also shown (Evans & Alexander, 1976) that reintroduction of the specific antigen will induce macrophages to kill tumour cells non-specifically, but not normal cells. However, the specific antigen cannot be a tumour-specific antigen, since the carcinogen is not introduced at the time of sham thymectomy. Repair after sham thymectomy would produce tissue breakdown followed by rapid growth of normal cells, so that perhaps "altered self" might provide the antigen to trigger T lymphocytes. Keller (1976) has shown that macrophages could be involved in the inhibition of growth of rapidly growing cells, and this involvement would then become thymus-dependent. Differentiation antigens (Old, 1977) might have become altered and thus immunogenic. Risser *et al.* (1978) have reported that with a virally induced lymphoma there is immunogenicity towards a normal differentiation antigen. Auto-immunity would act favourably in the surgically operated animals.

Alternatively, endogenous C-type RNA viruses may be activated by a variety of intrinsic factors (Todaro, 1975) and expressed during a period of growth (as in the repair after surgery). T cells may then respond to this expression on the proliferating cells, which may also be present on the mesothelioma cells. This would be a T-cell-dependent effective surveillance of virus expressed on tumour cells, although the mesothelioma is not necessarily virus-induced. Finally, whatever substance has produced specific macrophages, these cells can become non-specifically cytotoxic to tumour cells from lymphokines produced as a result of antigens produced during persistent infection (Hibbs *et al.*, 1972; Piessens *et al.*, 1975).

I would like to thank Mr G. Berry for much help and statistical advice, and Dr J. C. Wagner for continued help.

### REFERENCES

ALLISON, A. C. & TAYLOR, R. B. (1967) Observations on thymectomy and carcinogenesis. *Cancer Res.*, **27**, 703.

BALNER, H. & DERSJANT, H. (1966) Neonatal thymectomy and tumour induction with methylcholanthrene in mice. *J. Natl Cancer Inst.*, **36**, 513.

BERRY, G. & WAGNER, J. C. (1969) The application of a mathematical model describing the times of occurrence of mesotheliomas in rats following inoculation with asbestos. *Br. J. Cancer*, **23**, 582.

COX, D. R. (1972) Regression models and life-tables. *J. R. Statist. Soc.*, *B* **34**, 187.

EVANS, R. & ALEXANDER, P. (1972) Mechanism of immunologically specific killing of tumour cells by macrophages. *Nature*, **236**, 168.

EVANS, R. & ALEXANDER, P. (1976) Mechanisms of extracellular killing of nucleated mammalian cells by macrophages. In *Immunobiology of the Macrophage*, Ed. D. S. Nelson. London: Academic Press. p. 535.

GRANT, G. A. & MILLER, J. F. A. P. (1965) Effect of neonatal thymectomy on the induction of sarcomata in C57BL mice. *Nature*, **205**, 1124.

HIBBS, J. B. JR., LAMBERT, L. H. JR. & REMINGTON, J. S. (1972) Control of carcinogenesis: a possible role for the activated macrophage. *Science*, **177**, 998.

JOHNSON, S. (1968*a*) The effect of thymectomy and of the dose of 3-methylcholanthrene on the induction and antigenic properties of sarcomas in C57BL mice. *Br. J. Cancer*, **22**, 93.

JOHNSON, S. (1968*b*) Effect of thymectomy on the induction of skin tumours by dibenzanthracene, and of breast tumours by dimethylbenzanthracene in mice of the IF strain. *Br. J. Cancer*, **22**, 755.

KELLER, R. (1976) Cytostatic and cytocidal effects of activated macrophages. In *Immunobiology of the Macrophage*. Ed. D. S. Nelson. London: Academic Press. p. 487.

LAW, L. W. (1965) Neoplasms in thymectomized mice following room infection with polyoma virus. *Nature*, **205**, 672.

MILLER, J. F. A. P., GRANT, G. A. & ROE, F. J. C. (1963) Effect of thymectomy on the induction of skin tumours by 3,4-benzopyrene. *Nature*, **199**, 920.

NISHIZUKA, Y., NAKAKUKI, K. & USUI, M. (1965) Enhancing effect of thymectomy on hepatotumorigenesis in Swiss mice following neonatal injection of 20-methylcholanthrene. *Nature*, **205**, 1236.

NOMOTO, K. & TAKEYA, K. (1969) Immunologic properties of methylcholanthrene-induced sarcomas of neonatally thymectomized mice. *J. Natl Cancer Inst.*, **42**, 445.

NORTH, R. J. & KIRSTEIN, D. P. (1977) T-cell mediated concomitant immunity to syngeneic tumors. I. Activated macrophages as the expressors of non-specific immunity to unrelated tumors and bacterial parasites. *J. Exp. Med.*, **145**, 275.

OLD, L. J. (1977) Cancer immunology. *Sci. Am.*, **232**, 62.

PIESSENS, W. F., HALLOWELL, C. W. JR. & DAVID, J. R. (1975) Macrophages activated *in vitro* with lymphocyte mediators kill neoplastic but not normal cells. *J. Immunol.*, **114**, 293.

PIKE, M. C. (1966) A method of analysis of a certain class of experiments in carcinogenesis. *Biometrics*, **22**, 142.

POLLIACK, A., LEVIJ, I. S. & PFEFFERMAN, R. (1972) Observations on the effect of thymectomy on chemical carcinogenesis in the hamster cheek pouch. *Br. J. Cancer*, **26**, 368.

RISSER, R., STOCKERT, E. & OLD, L. J. (1978) Abelson antigen: a viral tumor antigen that is also a differentiation antigen of BALB/c mice. *Proc. Natl Acad. Sci. U.S.A.*, **75**, 3918.

RUSSELL, S. W., DOE, W. F. & MCINTOSH, A. T. (1977) A noncytolytic stage of macrophage activation in Moloney sarcomas. In *The Macrophage and Cancer*. Eds. K. James, B. McBride & A. Stuart. Edinburgh Univ. Medical School. p. 341.

TODARO, G. J. (1975) Evolution and modes of transmission of RNA tumor viruses. *Am. J. Pathol.*, **81**, 590.

WAGNER, J. C. & BERRY, G. (1969) Mesothelioma in rats following inoculation with asbestos. *Br. J. Cancer*, **23**, 567.

WAGNER, J. C., BERRY, G. & TIMBRELL, V. (1973) Mesotheliomata in rats after inoculation with asbestos and other materials. *Br. J. Cancer*, **28**, 173.

WAKSMAN, B. H., ARNASON, B. G. & JANKOVIC, B. D. (1962) Role of the thymus in immune reactions in rats. III. Changes in the lymphoid organs of thymectomized rats. *J. Exp. Med.*, **116**, 187.

YASUHIRA, K. (1969) Suspicious influence of thymectomy on skin papilloma induction. *Gann*, **60**, 57.